# Electrostatic subframing and compressive-sensing video in transmission electron microscopy

**DOI:** 10.1063/1.5115162

**Published:** 2019-09-23

**Authors:** B. W. Reed, A. A. Moghadam, R. S. Bloom, S. T. Park, A. M. Monterrosa, P. M. Price, C. M. Barr, S. A. Briggs, K. Hattar, J. T. McKeown, D. J. Masiel

**Affiliations:** 1 Integrated Dynamic Electron Solutions, Inc., Pleasanton, California 94588, USA; 2 Sandia National Laboratories, Albuquerque, New Mexico 87185, USA; 3 Oregon State University, Corvallis, Oregon 97331, USA; 4 Lawrence Livermore National Laboratory, Livermore, California 94551, USA

## Abstract

We present kilohertz-scale video capture rates in a transmission electron microscope, using a camera normally limited to hertz-scale acquisition. An electrostatic deflector rasters a discrete array of images over a large camera, decoupling the acquisition time per subframe from the camera readout time. Total-variation regularization allows features in overlapping subframes to be correctly placed in each frame. Moreover, the system can be operated in a compressive-sensing video mode, whereby the deflections are performed in a known pseudorandom sequence. Compressive sensing in effect performs data compression before the readout, such that the video resulting from the reconstruction can have substantially more total pixels than that were read from the camera. This allows, for example, 100 frames of video to be encoded and reconstructed using only 15 captured subframes in a single camera exposure. We demonstrate experimental tests including laser-driven melting/dewetting, sintering, and grain coarsening of nanostructured gold, with reconstructed video rates up to 10 kHz. The results exemplify the power of the technique by showing that it can be used to study the fundamentally different temporal behavior for the three different physical processes. Both sintering and coarsening exhibited self-limiting behavior, whereby the process essentially stopped even while the heating laser continued to strike the material. We attribute this to changes in laser absorption and to processes inherent to thin-film coarsening. In contrast, the dewetting proceeded at a relatively uniform rate after an initial incubation time consistent with the establishment of a steady-state temperature profile.

## INTRODUCTION

Transmission electron microscopy (TEM) is increasingly beset by data-throughput limitations, dictated in part by readout rates of TEM cameras. Part of the reason is the rise of TEM techniques yielding data of more than two dimensions, with additional dimensions including time (dynamic, ultrafast, and *in situ* TEM), tilt angle or depth (tomography), energy (electron energy loss, energy-dispersive x-ray, and cathodoluminescence spectrum imaging), and two-dimensional scattering angles [the field most generally called 4D-STEM, including scanning TEM (STEM) diffraction, orientation imaging, electron ptychography, and some forms of fluctuation electron microscopy].^1–7^ These operating modes demand extreme data throughput and can be severely limited by a given instrument's data capture bottleneck.

In particular, *in situ* TEM is an excellent platform for studying nanoscale material processes, but often these processes are too fast for conventional TEM cameras. Prime examples include pulsed-laser modification of nanoscale materials, including sintering and dewetting of nanoparticle aggregates and coarsening of nanocrystalline material, all of which are relevant to technological applications. Pulsed-laser sintering of particle aggregates, for example, is a core process for many additive manufacturing technologies.^8^ There are extremely few tools for monitoring this process on its natural length and time scales. Movie-Mode Dynamic TEM (MM-DTEM)^4,9^ offers burst-mode multimegahertz TEM imaging frame rates, but only a small number of such instruments exist, and the number of frames per event is currently limited to 16. Multikilohertz high-frame-count TEM video acquisition would therefore be an essential enabling technology for a large and timely class of nanoscale materials dynamics studies. Lacking such tools, studies are often limited to microstructural before-and-after comparisons and are blind to the crucial transient states that explain “why” the microstructure changed in the way that it did.

This need is well recognized in the TEM community, evidenced by the rapid recent development of high-speed cameras with sustained frame rates of hundreds or even thousands of frames per second.^1,2,10–15^ These cameras are typically direct-detection systems not subject to the physical time-resolution limits of older indirect scintillator/CCD-based cameras. This allows sustained high frame rates to be achieved through scaling and optimization of the entire digital data pathway starting with the pixel readout. When the digital throughput limit is reached, most cameras allow users to further increase speed by trading off the frame rate against the pixel count per frame, through hardware binning or by reading out a selected subregion of the camera. These options vary with camera design, but this trade-off is typically on a one-for-one basis (i.e., the total number of pixels per second is constant) or less.

**FIG. 1.**
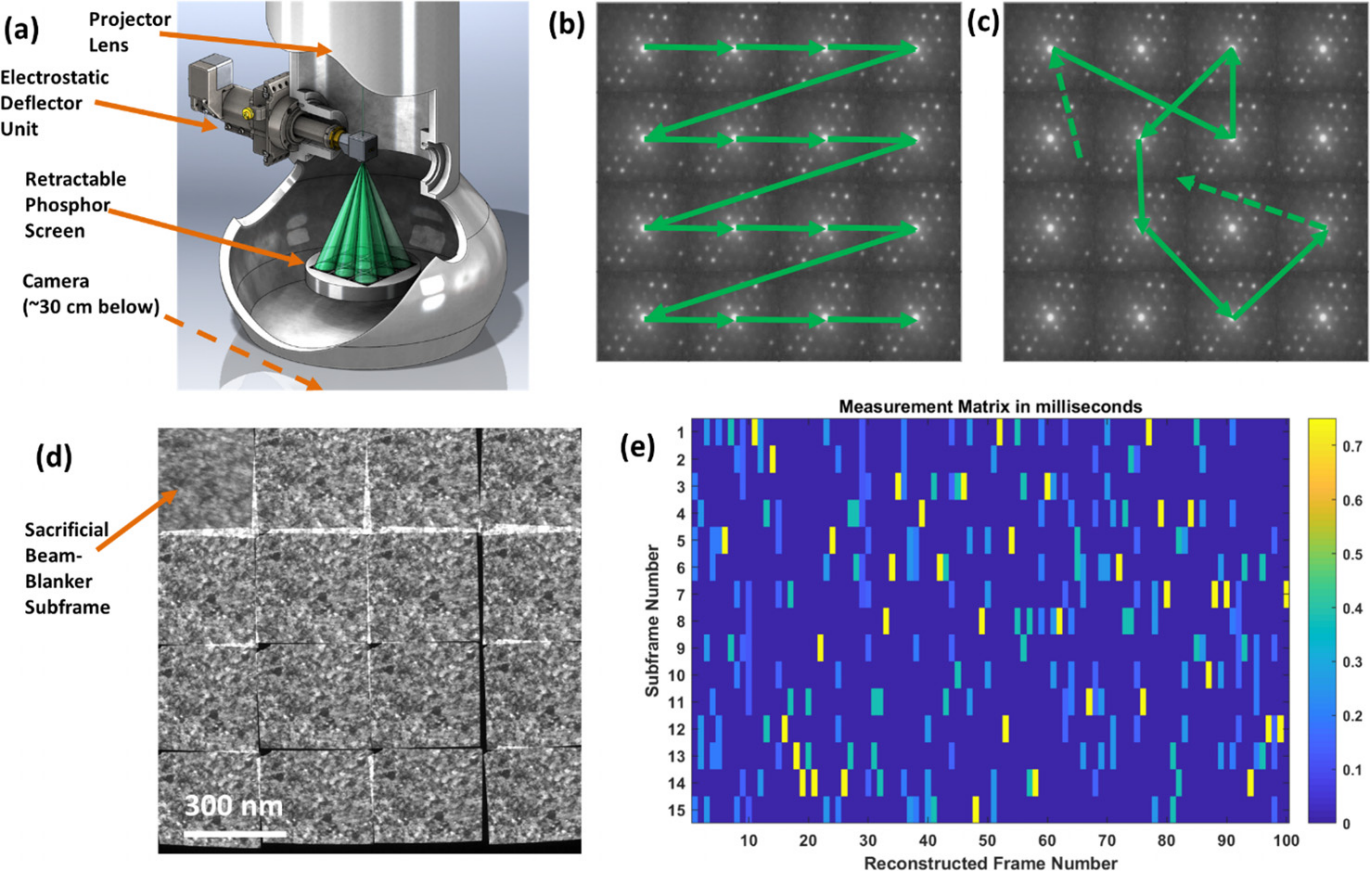
General illustration of electrostatic subframing (ES) in a TEM. (a) A fast two-dimensional electrostatic deflector is inserted below the projector lens and well above the camera, allowing a square array of subframes to be defined (angles exaggerated for illustration). (b) A hypothetical image that could be captured in a single acquisition in a 4 × 4-subframe mode (with TEM in diffraction mode) and using 1-to-1 sequential subframing, producing 16 diffraction patterns from one camera acquisition. (c) The CS mode replaces the 1-to-1 deflection sequence with a pseudorandom sequence shifting subframes multiple times per time slice, with typically 100 time slices and ∼500 subframe transitions defined per camera exposure time. CS algorithms then allow 100 frames to be reconstructed from one exposure. (d) A typical measurement of a static sample (nanocrystalline platinum, acquired on the SNL I^3^TEM). Each subframe except for the upper-left sacrificial subframe is a 12 ms exposure. (e) An example temporal measurement matrix *M*, showing how time is apportioned to each subframe during each time slice in pseudorandom mode. In this example, one sacrificial beam-blanker subframe was removed, leaving 15 for the main acquisition, thus 15 rows in the matrix. 100 time slices were used in this example (number of columns in the matrix).

Here, we present a complementary approach to time resolution through a completely different mechanism that we call electrostatic subframing (ES) (Fig. 1). An electrostatic deflector with a nominally square limiting aperture is placed just after the TEM's projector lens (the last electron-optical lens in the standard microscope column) to allow a TEM camera to be subdivided into a square array of subframes. The deflector determines which subframe is exposed at each moment. The ES system presented in this article can produce subframe array sizes from 2 × 2 up to 16 × 16. The results we present use a 4 × 4 array size.

ES can augment the performance envelope of a camera in three distinct ways. First, in a continuous acquisition mode, it allows the one-for-one trade-off curve of pixel count against the frame rate to be extended beyond the range normally available to the camera. ES allows a 16-megapixel camera to act like a 1-megapixel camera with 16 times the frame rate or a 0.06-megapixel camera with 256 times the frame rate, for example. Second, in a burst acquisition mode, ES completely decouples the time resolution from considerations such as phosphor decay times, beam blanker settling times, and readout times by combining multiple frames of the video into a single acquisition. Third, ES enables compressive sensing (CS), whereby the reconstructed video can contain substantially more pixels or subframes than were read in from the camera. For example, in the 4 × 4 ES tests presented below, we demonstrate the ability to reconstruct not 16 but 100 subframes of videos from one burst-mode camera acquisition, i.e., a frame rate compression ratio of more than 6:1. In the present article, we will illustrate the burst-mode and CS advantages by demonstrating the ability to record known laser-driven material processes at kilohertz-scale frame rates using a TEM camera normally limited to subhertz frame rates. As this is the first detailed introduction of the ES/CS method, we confine ourselves to demonstrations of already-known processes in order to validate the approach. Continuous-mode acquisition will be discussed in a separate publication.

CS is a set of approaches for applying data compression to the acquisition of data rather than just the transmission and storage of data,^16–20^ by operating the equipment in a nonstandard way that effectively performs data compression before the signal reaches the analog to digital convertors. We now summarize the core points of the mathematical theory of CS, established in seminal papers from over a decade ago^16–19^ and actively researched to the present day in many areas including electron microscopy.^20–30^ The theory shows that an *N*-dimensional vector *x* may be recovered to within specified error bounds with *K *<* N* measurements *y* if (1) *x* is known to be sparse in some known or discoverable mathematical representation defined by a “sparsifying transform” *W*, (2) *K* is sufficiently large, and (3) the CS measurement matrix that controls how the elements of *x* are mixed and subsampled to produce *y* is well chosen. Further, this recovery may be performed efficiently such as by regularizing the underdetermined measurement equations by penalizing the *l*_1_ norm applied to the sparsified data, i.e., |*Wx*|_1_. Subject to some minimal assumptions, the convex nature of the optimization problem ensures that the algorithms will converge to an optimal solution with error bounds guaranteed to high probability.^16–19^

Various efforts have been made to apply CS and related concepts (such as undersampling and inpainting) to electron microscopy.^21–30^ These efforts demonstrate that conventionally acquired electron microscopy data are often heavily oversampled relative to their true information content and that acceptable results are often possible even with compression ratios *N*/*K* of ∼10 or more.

**FIG. 2.**
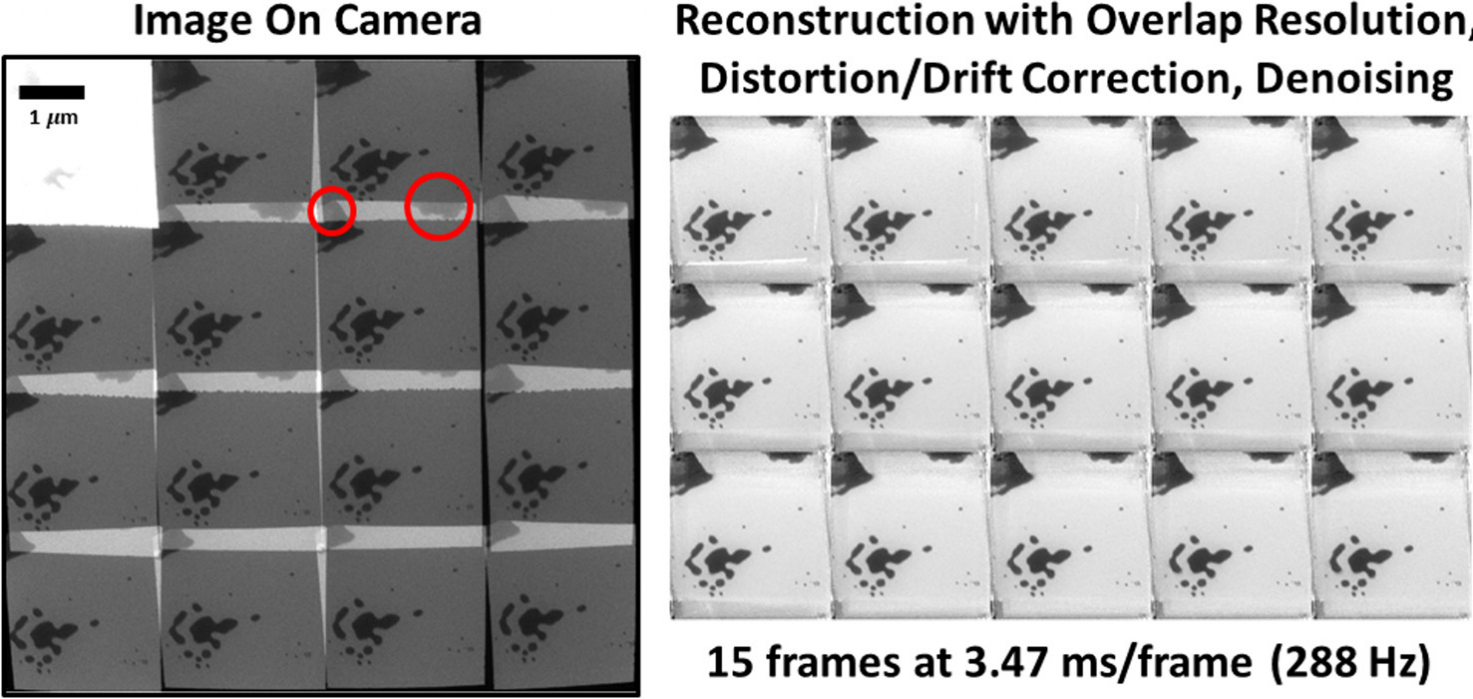
1-to-1/OR reconstruction of melting/sintering of nanostructured Au on SiN_x_. The raw camera image was preprocessed to eliminate the upper-left sacrificial frame and then segmented, aligned, and denoised using the same algorithm used for CS reconstructions to produce the overlap-resolved, drift-stabilized 15-frame video. Circles highlight features relevant to the overlap resolution; see the text. See also Fig. S2 and Videos S2 and S3.

ES enables compressive video acquisition in two distinct modes. First, we consider the overlap resolution (OR) in what we call “1-to-1” sequences [Fig. 1(b)] such that each subframe is exposed in a simple sequence, once per camera readout. OR can yield modest compression ratios in terms of reconstructed video pixels per original-image pixel. Very often, the subframes in an ES acquisition will overlap [Figs. 1(d) and 2(a), for example], with some pixels receiving information from two or more subframes. Typically, the deflector will be configured to make the best use of the camera area, with the great majority of pixels belonging to one and only one subframe. Yet, one may also configure the system to produce a substantial overlap, as desired. As we will show, the information in these overlap regions is not lost; a CS-inspired reconstruction algorithm can successfully determine which features belong to each of the overlapping subframes. It is this mechanism—enabling one camera pixel in an overlap region to inform two or more output video voxels—that enables compression ratios greater than one in this mode. The 1-to-1/OR mode is worth considering when the signal-to-noise ratio and/or the compressibility of the underlying data stream would not support more aggressive compression techniques. We shall present a proof-of-principle result from this operating mode, in which nanoparticle aggregates in the overlap regions are clearly identified as coming from the correct subframes, and 15 frames of the overlap-resolved video are recovered from a single camera acquisition.

The second compressive video acquisition mode is a true temporal CS video mode, in our tests offering frame-count compression ratios of 6.67 (i.e., 100 frames of video reconstructed from 15 subframes). The deflector is not operated in a 1-to-1 sequence but in a known pseudorandom sequence, distributing the intensity in each short span of time among several of the subframes [Fig. 1(c)]. The sequence is dictated by a temporal measurement matrix *M* [Fig. 1(e)]. The rows of *M* correspond to subframes on the camera, while the columns represent equal subdivisions—which we term “time slices”—of the “effective exposure time” during which the electron beam is striking the camera (corrected for the measured speed of the TEM's beam blanker).

The time slices are defined as the finest temporal resolution at which we will attempt reconstruction. This means that all points in time within a single slice are considered simultaneous. The entries in the matrix are dwell times, in milliseconds. Thus, in the example in Fig. 1(e), during the first time slice, the deflector spends equal time in subframes 3, 5, 6, and 15 (the row numbers in which the first column is nonzero). During the second time slice, the deflector partially exposes subframes 4, 12, and 13, and so forth, so that the sum of all elements in one column of *M* is the duration of a time slice and the sum of all elements is the total effective exposure time for the entire camera frame. The matrix elements can be specified arbitrarily, apart from non-negativity and sum constraints, thus allowing our approach to enter the domain of the classic results in the CS literature^16–19^ which demand the measurement matrix to have certain mathematical properties, including incoherence with respect to *W*. In the large-*N* limit, Bernoulli random measurement matrices generally have these properties to high probability for many choices of *W*.^16–19^ We therefore used Bernoulli random matrices for the test results presented here. Because the measurements are dominated by noise that scales as Poisson noise (see Materials and Methods), the optimal selection fraction for the Bernoulli matrices is less than 0.5;^29^ we set this parameter in the range of 0.15–0.35 for most of our tests.

Each subframe is exposed with a different temporal multiple-exposure of the series of events occurring during the entire exposure time. A computer can then, knowing the camera image, the *M* matrix, and the sparsity-inducing transformation *W*, determine the sparsest video sequence consistent with the measurement. As we may specify *M* and *W* arbitrarily, we can take advantage of the voluminous CS applied mathematics literature for performing robust reconstructions. Using this form of CS video, we have reconstructed 50- to 100-frame videos from single camera acquisitions, with effective frame rates ranging from 500 Hz to 10 kHz. This was performed on conventional scintillator/fiber-bundle/CCD TEM cameras normally limited to subhertz frame rates.

## RESULTS

Initial proof-of-principle tests were performed on the Dynamic Transmission Electron Microscope (DTEM) facility at the Lawrence Livermore National Laboratory (LLNL),^4,9^ while the bulk of the results were generated using the *In situ* Ion Irradiation TEM (I^3^TEM) at Sandia National Laboratories (SNL), Albuquerque, equipped with a thermionic LaB_6_ electron source.^31^ An additional system is installed at the Ultrafast Dynamic Transmission Electron Microscope (UDTEM) facility at the University of Strasbourg.^32^

Since the first tests (Fig. S1 and Video S1), we have improved the voltage-driving circuits, control systems, and data analysis methods and are now able to produce much higher quality video reconstructions of real *in situ* experiments. Figure 2 (along with Fig. S2 and Videos S2 and S3) is a prime example. This measurement shows a 1-to-1/OR acquisition of laser-driven melting and dewetting of presintered (via previous laser shots) gold nanoparticle aggregates on a silicon nitride substrate, using the I^3^TEM. To the left of Fig. 2, we see the raw image on the camera, with 16 subframes superposed in a single camera exposure. The overexposed upper-left frame is a beam-blanker sacrificial frame that is removed from the analysis; see Materials and Methods. The remaining 15 frames thus constitute the time-resolved measurement, with the 52 ms effective exposure time parceled equally into each subframe in sequence. The frame rate is thus (15 frames)/(52 ms) = 288 Hz (i.e., 3.47 ms/frame). The 1.06 *μ*m sample drive laser, running on its own 33 kHz clock and producing 36 ns FWHM pulses with a diameter of approximately 100 μm at the sample, is gated by a signal sent by the same digital sequencer that controls the deflector. Thus, the laser is on only during the 52-ms reconstructed exposure time devoted to the 15 frames (i.e., not including the beam-blank sacrificial time; again, see Materials and Methods). Photodiode oscilloscope measurements show that the sample drive laser takes ∼0.5 ms to ramp up (Fig. S5), after which the pulse intensities reach the steady-state and remain there throughout the exposure. The same temporal profile is used for all laser-driven experiments.

The data analysis, detailed in Materials and Methods, produced the 15-frame reconstructed video while simultaneously compensating for multiple effects: the displacement and distortion of each subframe on the camera, camera noise and other sources of error, sample drift, subframe overlap on the camera, and CS temporal mixing. The combination of these factors lets the algorithm determine a calibrated $\chi^{2}$ per pixel for a given reconstruction estimate *x*. It then iterates, finding the target solution that minimizes anisotropic total variation (TV; defined in Materials and Methods) for a given $\chi^{2}$/pixel target κ. In other words, it finds the reconstructed video that is simultaneously (1) consistent with the measurement, quantitatively accounting for all known sources of distortion and error, to a precision specified by κ and (2) has as low as possible a value of *TV* = ${\left|\mathrm{Wx}\right|}_{1}$, where *W* is a scaled discrete gradient operator. Much previous work has shown TV-minimization to be an effective choice for regularizing noisy and underdetermined video reconstruction.^33^ More advanced methods, e.g., using total generalized variation (TGV), dictionary learning, etc., also exist,^30,34,35^ but for the present results, we found more than adequate performance using simple anisotropic TV minimization. The algorithms could of course be generalized as dictated by the results of further experimental tests.

**FIG. 3.**
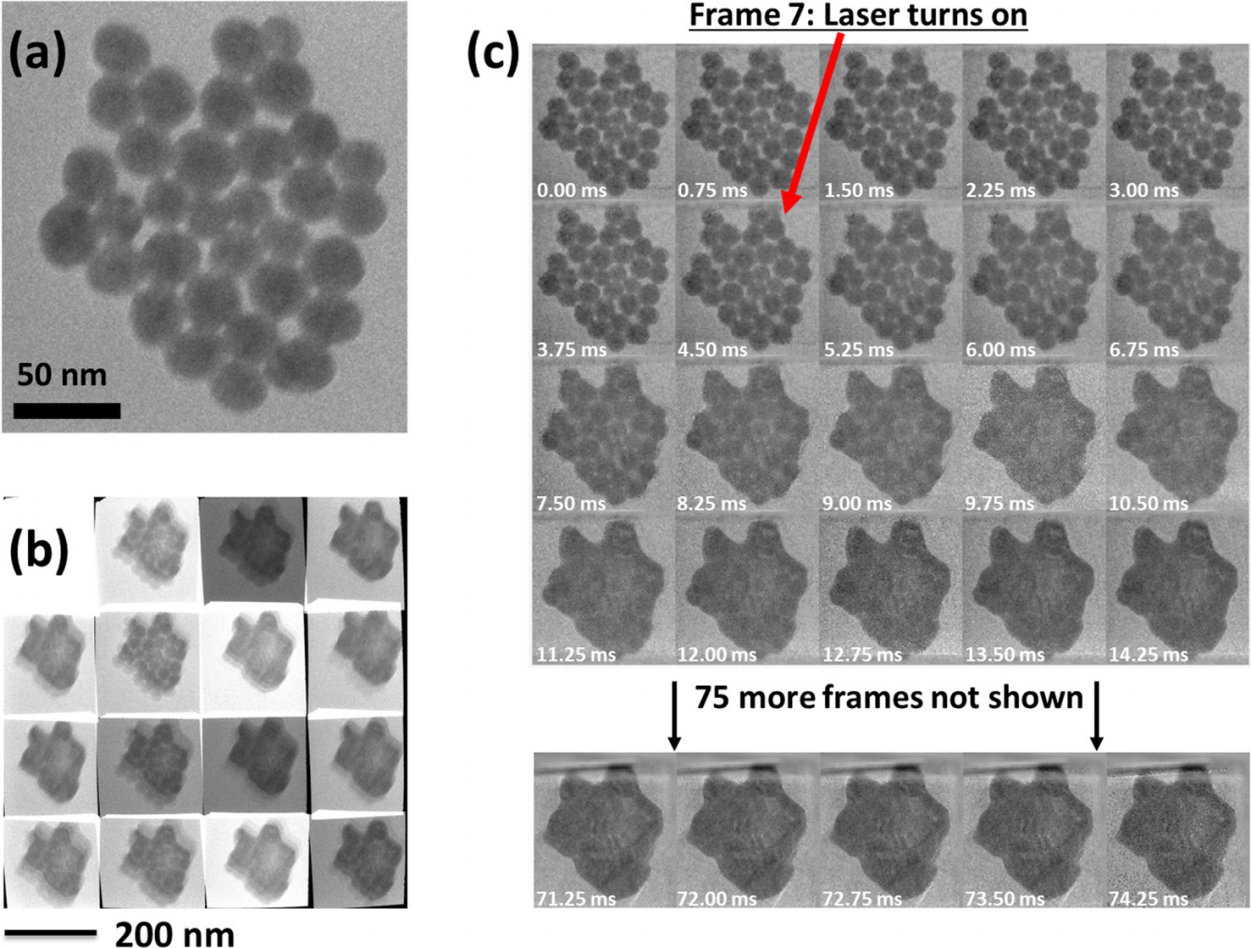
1.33 kHz 100-frame CS video reconstruction from a single TEM camera exposure of laser sintering of Au nanoparticle aggregates. (a) Initial state; a single isolated cluster of Au nanoparticles on SiN_x_. (b) Raw image on the camera, with contrast set to accentuate the 15 nonsacrificial frames at the cost of the upper-left sacrificial frame and overlap regions. (c) 100-frame video reconstructed from the single measurement in (b).

We contrast the 1-to-1/OR-mode measurement of melting/dewetting with another measurement, this time laser-sintering an isolated aggregate of ∼20-nm gold particles [before-shot state in Fig. 3(a)] that had not been sintered by previous laser shots (Figs. 3 and S3 and Videos S4 and S5). This exposure was performed in the pseudorandom CS mode [Fig. 1(c)], using a temporal measurement matrix *M* similar to that in Fig. 1(e), with 100 frames of video superposed into 15 subframes during a single camera acquisition [raw camera image in Fig. 1(b)]. The video covers a total of 75 ms, thus yielding a reconstruction with 750 μs per frame, i.e., a 1.33 kHz effective frame rate. The first 20 and last 5 reconstructed frames, zoomed in to highlight the region of interest, are shown in Fig. 3(c). We deliberately delayed the onset of the laser to frame 7, in order to establish the initial state and to test for possible “negative time” artifacts during the CS reconstruction (i.e., sample evolution appearing to start before the sample drive laser fires, which would be indicative of an incorrect reconstruction). This test verified that the reconstruction produced no such artifacts; the first 6 reconstructed frames are essentially identical. The laser, once activated, remained on until the end of the 75 ms effective exposure time. After a short (roughly 2–3 ms) incubation time, the sample was hot enough to initiate sintering. Most of the sintering was complete within another 3–4 ms; we can see that the aggregate has contracted slightly and the interstitial spaces between the nanoparticles have been mostly filled in. Little evolution occurs after this time, as is evident in comparison between the frames at 14.25 and 74.25 ms.

**FIG. 4.**
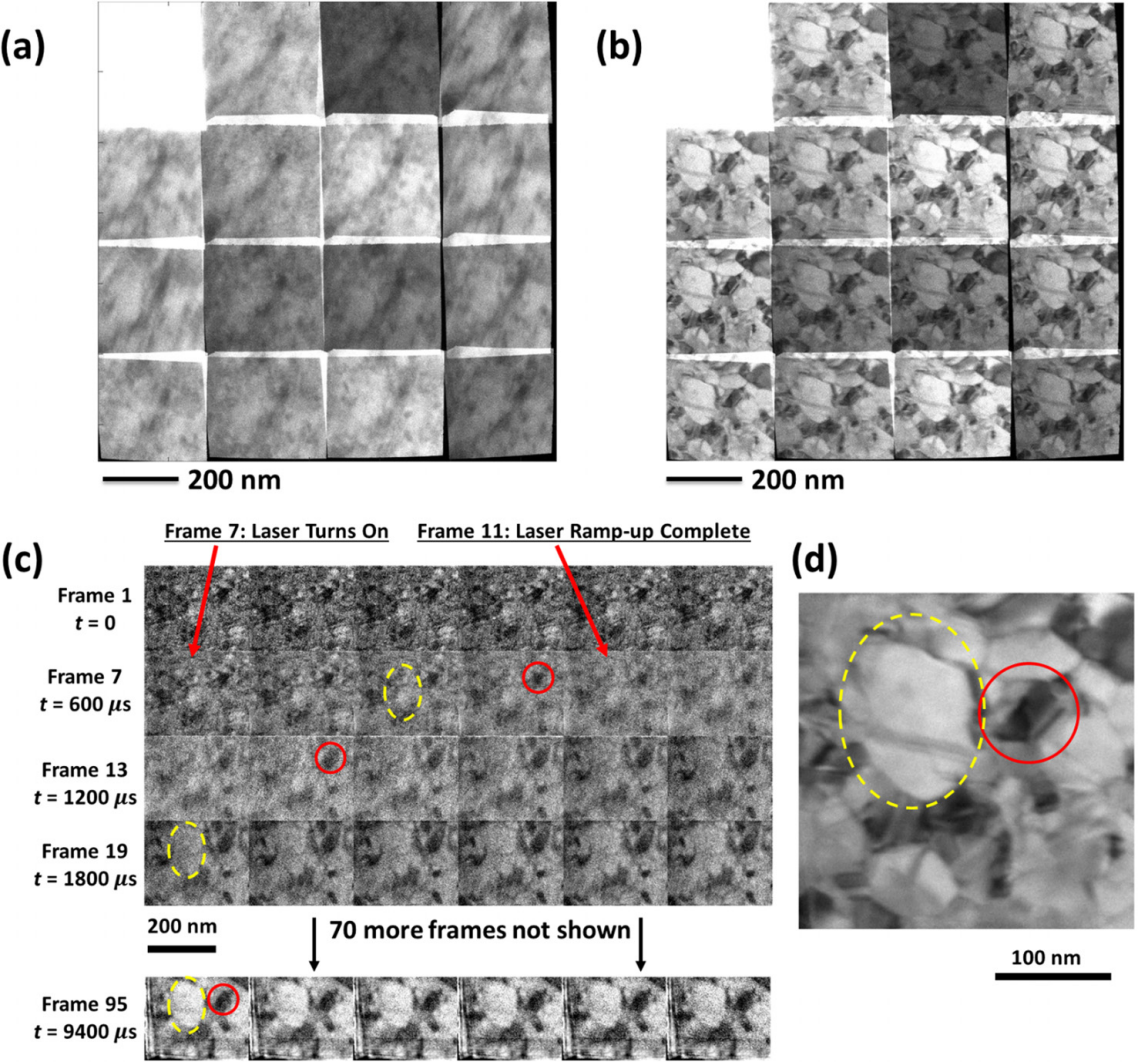
10 kHz CS video of laser-driven coarsening of nanocrystalline Au. (a) Raw measurement of time-resolved video. Contrast range set to saturate the upper-left sacrificial frame and thus accentuate contrast in the remaining 15 frames, totaling 10 ms. (b) Similar raw measurement of the postlaser-shot static coarsened sample. (c) Selected frames of the 100-frame CS video reconstruction derived from (a). Notable features accentuated with red circles and yellow dashed ellipses. (d) Single-frame static reconstruction derived from (b), representing an aligned, denoised combination of 15 subframes with a total exposure time of 10 ms. Circles (red in online version) and dashed ellipses (yellow in online version) denote the same features as in (c).

We provide a final example, again involving laser-driven modification of nanostructured gold, but this time in the form of grain coarsening of a 50-nm-thick nanocrystalline thin film (Figs. 4 and S4 and Videos S6 and S7). Grain coarsening, one of the fundamental processes of metallurgy, is a process, whereby crystallographic grain boundaries move, causing some grains to grow at the expense of others, and the average grain size increases as the smallest grains are consumed. Knowing this to be potentially a very fast process, we reduced the exposure time to 10 ms while keeping the number of reconstructed frames at 100; thus, the reconstructed video has a frame rate of 10 kHz, i.e., 100 μs per frame. This was a deliberate attempt to identify the performance limits of temporal compressive sensing, with the study of a relatively low-contrast very-fast-evolving sample at extreme frame rates. Again, we started the sample drive laser in frame 7 and, again, we verified the lack of negative-time artifacts in the first 6 frames.

While the signal-to-noise ratio in the 10 kHz reconstruction is low, we can focus on the most persistent, high-contrast features such as the small dark grain circled in red in Fig. 4. Before the laser pulses arrive, this grain appears roughly equiaxed with a diameter of approximately 30 nm. At the end of the experiment, after the sample has cooled [Fig. 4(d)], this grain has roughly doubled in diameter while taking on an elongated, faceted shape. The reconstructed video reveals that this grain had very nearly its initial size and shape in frame 10 (near the end of the 500 μs drive laser ramp-up) and very nearly its final size and shape by frame 14, a mere 400 μs later. It continues to evolve, its contrast darkening and its edge sharpening, over the next several frames but shows relatively little further change after roughly frame 20. Similarly, the large ∼100-nm-diameter grain visible to its left (highlighted with yellow dashed ellipses) appears to grow from a roughly 50-nm-diameter precursor to very nearly its final size over essentially the same span of time. In fact, apart from gross thermal sample motion and a general increase in contrast and edge sharpness, the great majority of evolution is confined to a 1-ms span starting near the end of the laser ramp-up in frame 11. This is remarkable, considering that the sample-drive laser continued to strike the sample at its maximum intensity for the balance of the 10-ms exposure time. We return to this observation in the Discussion section.

The postshot static image [Fig. 4(d)] in this experiment illustrates one final operating mode for the ES system: alignment, noise-weighted averaging, and optional denoising of multiple short-exposure dose-fractionated images. This mode is appropriate for radiation-damage-tolerant imaging of damage-sensitive materials, an extremely important topic in modern TEM. The raw image [Fig. 4(b)] was captured immediately after the 10 kHz time-resolved video, with the system parameters unchanged except for deactivation of the sample drive laser trigger/gate signal. Thus, the total exposure time was again 10 ms, but the sample was static. Exactly the same analysis procedure was performed, except we instructed the software to merge all 100 time slices into a single binned time slice, i.e., we asked it to reconstruct a single frame optimally combining information from all 15 subframes. The formalism automatically accounted for the different exposure times, shifts/distortions, and noise levels in the 15 frames and produced the appropriate TV-denoised weighted average.

## DISCUSSION

Three aspects of the results must be discussed: (1) the features of the ES method, its various operating modes, and the reconstruction software; (2) the scientific interpretation of the experiments, comparing and contrasting the three very different processes under study in laser-driven nanostructured gold; and (3) potential future development of ES and its applications to *in situ* TEM and multidimensional TEM in general.

### Features of the ES method revealed by the results

We have shown how ES can resolve millisecond-level or faster details in *in situ* TEM experimentation, using conventional TEM cameras normally limited to subhertz operation. The 4 k × 4 k camera used on the I^3^TEM, for example, takes multiple seconds to read out a full frame in low-noise mode and thus is not even capable of 1 Hz operation at maximum image quality; yet, it produced video recordings up to 10 kHz. All the tests produced results that could not have otherwise been obtained with the available camera. The system can be precisely synchronized with both the TEM camera and external *in situ* sample drive capabilities such as pulsed lasers, electrical biasing, and mechanical loading and indenting. We have demonstrated three distinct operating modes: 1-to-1 mode with an optional overlap resolution, pseudorandom compressive sensing mode, and single-frame reconstruction of a static sample. All modes allow dose fractionation and time resolution on time scales inaccessible to most TEM cameras. Further, the system decouples the time resolution from the camera properties, allowing continuous adjustment of frame rates from subhertz to multikilohertz rates through simple software settings.

The same CS reconstruction software is used with all three modes (see Materials and Methods). It features automatic compensation for distortion, calibrated camera noise allowing a true $\chi^{2}$ per pixel target to be specified (thus avoiding both overfitting and oversmoothing of the data in a statistically justifiable way), overlap resolution, iterative sample-drift correction, and adjustable anisotropic TV denoising of the result.

The overlap resolution in 1-to-1/OR mode is apparent in Fig. 2. Two large aggregates are clearly visible in subframe-overlap regions in the raw data (red circles). A human observer can easily see that these aggregates are in the upper parts of the frame, not the lower parts, even in the case of the upper-right-hand aggregate (the image of which is almost entirely confined to the subframe-overlap regions on the camera). The TV-minimizing algorithm had no difficulty correctly assigning these features to the correct frames; these aggregates appear only in the upper parts of the 15 reconstructed frames and not the lower parts.

Note that the upper-right aggregate only appears clearly in a few subframes of the raw measurement; it moves out of frame due to the large-scale motion of the sample caused by the localized heating of the sample drive laser. Yet, it appears in all 15 frames of the reconstruction. This is implicit in the three-dimensional TV regularization. Lacking information to the contrary, the algorithm will populate unobserved space-time voxels in the reconstructed video with intensities from the nearby observed voxels. In effect, when a part of the aggregate moves out of frame, the algorithm remembers what it last looked like and makes the simplest assumption—namely, that it did not change since then. The algorithm is thus doing exactly what we asked it to do: Make its best guess of the “simplest” (least-TV) video consistent with the measurement, using all available data. Something similar happens near the bottom of the frame, where the algorithm tries (with limited success) to make sense of the imperfectly corrected subframe-edge artifacts. If a user is uncomfortable with these extrapolations, it is very easy to remove them at the end. The supplementary material includes reconstructed videos with and without this optional postprocessing; this is why the videos all have “All Voxels” and “Measured Voxels” versions (Figs. S2–S4 and Videos S2–S7). For most scientific applications, this restriction to measured voxels is likely to be the more appropriate choice, but in the present article, introducing the method, we have elected to present both of them.

All the videos have residual artifacts derived from the imperfect correction of the subframe edge effects as well as slight background-intensity variations especially near the edges of the frames. The subframe edges yield obvious thin nearly straight lines near the periphery of the reconstruction, varying discontinuously from one frame to the next. However, a human viewer can easily recognize and discount these, paying attention to the persistent nonartifact features, and this raises hopes that future algorithms may be able to eliminate them entirely. The background-intensity variation effect appears to come from nonideal behavior in the camera itself; the normalized-residual images associated with the diagnostic plots (Fig. S6, for example) very often are biased slightly positively in the columns containing the (heavily exposed) sacrificial subframe and slightly negatively in the columns far from this subframe. This may be a CCD-bleeding or similar artifact that causes nonlocal difficult-to-correct slight background shifts. In our judgment, given the apparent quality of the reconstructed videos, these artifacts are insufficient to invalidate the scientific interpretation of the results.

**FIG. 5.**
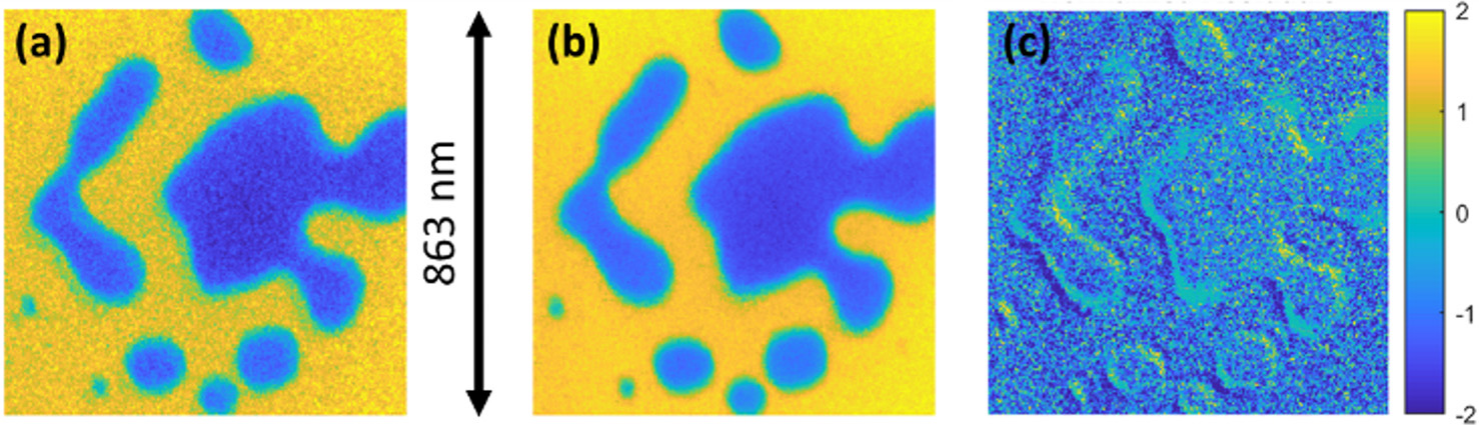
Details from Fig. 2, exemplifying the denoising/deblurring process. (a) Selected region from the measured image. (b) Corresponding region from the reconstruction, with denoising and motion-blur correction. (c) Normalized residuals [i.e., difference between (a) and (b) in units of RMS noise, after correction for distortion].

The reconstruction algorithms implicitly include denoising with quantitative consideration of the known noise in the system. This is illustrated in Fig. 5, a close-up of one of the frames of video from the 1-to-1/OR measurement in Fig. 2. The raw and reconstructed images are shown, alongside the normalized residual image (i.e., the difference between the two, in standard deviation units). We chose a subframe showing motion blur in the raw image in order to demonstrate how the algorithm can also correct for this. Random-looking speckle in both the features and the smooth-substrate background is greatly reduced in the denoised image. The nanoparticle edges are hardly touched apart from some reduction of intraframe motion blur visible as a weak ghost image superposed on the measured image, shifted slightly down and to the left. The sample drift correction allows for small rigid translations of each reconstructed frame to optimally align it to its neighbors. The reconstruction can then use the immediately preceding and following frames to help determine whether, for example, a blur artifact is likely to represent a real, persistent object. Users uncomfortable with this feature can turn it off by setting the temporal TV coefficient $\lambda_{t}$ to zero (see Materials and Methods). This allows the user to maximize the effective time resolution at the cost of increasing the noise in the result.

The drift-corrected TV-minimizing algorithm does this sort of denoising and deblurring implicitly, while also tending to sharpen the noisy edges of real features. In essence, we are telling the algorithm: If it is possible to make subtle adjustments to the pixel intensities, on the order of ±κσ (where κ is typically set to ∼1 but is adjustable, including to a low enough level that the denoising is essentially shut off), to eliminate local single- or few-pixel intensity maxima and/or minima, then do so. But if eliminating a local feature incurs a substantial χ^2^ penalty, then the feature should be retained. Note that we are using a 3D TV penalty, and if the same borderline-significant feature appears in multiple frames, it is more likely to be retained. Thus, neighboring-frame information is used to inform the decision in a statistically reasonable way. This adjustable high-performance statistically justifiable denoising is a feature for users who want it, and it can be essentially shut off for users who do not. Had we not calibrated the camera noise, it would be much more difficult to objectively justify the level of denoising; it would have to be adjusted according to a user's subjective judgment. But because we included the absolute noise calibration in the reconstruction algorithm, we achieve this calibrated denoising capability at essentially zero additional computational cost. The same calibrated denoising capability can also be used for non-ES conventional TEM measurements as well as in dose-fractionated static ES images such as in Fig. 4(d), derived from Fig. 4(c).

The drift and distortion corrections worked as intended, as is evident especially in Video S3, showing only the measured voxels in the 1-to-1/OR acquisition. The locations of the features of interest remain steady across the field of view (FOV) throughout the measurement, with residual uncorrected motion typically on the order of one pixel, as the outer edge of the field of view substantially distorts and shifts in every single frame. This frees the viewer to focus on the elements of scientific interest: The evolution of the sample itself, in its own rest frame. The distortion correction is robust, precise, and built into the reconstruction algorithm. The drift correction is similarly effective but, as with many features of the analysis, optional.

### Scientific interpretation of the measurements

We have briefly described the principle observations under Results. Here, we delve into the details in order to understand what the various ES operating modes can tell us about these three very different physical processes: melting/dewetting, sintering, and grain coarsening, all in laser-driven nanostructured gold.

We start with a discussion of time and length scales, especially those associated with diffusive thermal transport. These experiments incorporate multiple time scales covering a very large range, summarized in Table I. The thermal diffusive transport time scales are calculated for various characteristic size scales in each experiment, using thermal diffusivities *D* of 1.5 × 10^−6^ m^2^/s for silicon nitride thin films^36^ and 1.1 × 10^−4^ m^2^/s for gold.^37^ The melting/dewetting and sintering experiments were performed using isolated clusters of nanostructured gold on silicon nitride substrates, and thus, the diffusivity of gold is relevant for smaller scales and that of silicon nitride for larger scales. By field of view (FOV), we mean a region relevant to the experiment. For melting/dewetting and coarsening, this was the entire region visible in the video, while for the sintering experiment, we focus just on the nanoparticle cluster.

**TABLE I.** Time scales for the three example experiments. Time scales include both scales under direct experimental control (laser repetition rate, laser pulse duration, and reconstruction time slice, i.e., time for one reconstructed frame) and scales associated with thermal diffusion across typical features, fields of view (FOV), and the size of the laser-drive spot on the sample. Size scales are converted to time scales using $t\;∼\;\frac{L^{2}}{2D}$ with *D* being the thermal diffusivity. Note that the laser ramp-up time is ∼500 μs in all cases, i.e., the laser takes approximately 15 pulses to reach its steady-state peak power.

Experiment	Laser rep.	Laser pulse	Frame	Size scales			Time scales		
				Feature	FOV	Laser	Feature	FOV	Laser
Melting/dewetting	30 μs	36 ns	3.47 ms	∼100 nm Au	2.4 μm SiN_x_	∼100 μm SiN_x_	45 ps	1.9 μs	3 ms
Sintering	30 μs	36 ns	0.75 ms	∼20 nm Au	180 nm Au	∼100 μm SiN_x_	1.8 ps	150 ps	3 ms
Coarsening	30 μs	36 ns	0.10 ms	∼10 nm Au	300 nm Au	∼100 μm Au	0.45 ps	410 ps	45 μs

Table I clarifies several important facts. The thermal diffusivity of gold is so high that transport across a single nanoparticle, a crystalline grain, or even the entire field of view (for the sintering and coarsening experiments) is nearly instantaneous for our purposes. Thermal diffusion through the thickness of the 50 nm SiN_x_ support layers in the melting/dewetting and sintering experiments has a characteristic time scale of 0.8 ns, much shorter than the 36 ns laser pulse duration. The sample drive laser fires at 33 kHz at an intensity sufficient to raise gold to its melting point on a time scale on the order of a few milliseconds, depending on the experiment, and thus, each pulse raises the temperature by perhaps 20 K or less. Each laser pulse lasts 36 ns but is nonuniform in time such that the peak heating rate should be on the order of a few Kelvin per nanosecond, again depending on the experiment. Thermal diffusion (from Table I) and electron-phonon equilibration times within the gold are on the picosecond scale. All of this is consistent with peak local temperature-variation excursions on the order of a few Kelvin at most.

For the melting/dewetting experiment, we expect the laser absorption to be rather strong in the nanostructured gold relative to the silicon nitride support, and the absorption per volume in gold nanoparticles is strongly dependent on the size, surface curvature, and sizes and shapes of any gaps between nanoparticles which can form plasmonic “hot spots.”^38,39^ Thus, different nanoparticle clusters may be at different temperatures at the end of each laser pulse, but thermal conduction through the support should essentially erase this temperature difference on a time scale of 1.9 μs across the FOV used in the experiment, which is much less than the 30 μs gap before the next laser pulse and very much less than the 3.47 ms frame time and the ∼7 ms lag between the start of the laser pulse and the start of the motion. After this lag, the motion initiated on all nanoparticles in the field of view simultaneously and proceeded throughout the measurement at a nearly constant rate. The obvious interpretation is that it took the accumulated absorbed heat from roughly 200 laser pulses to melt the gold and that the thermal transport time scales are fast enough that, at the 288 Hz frame rate, the melting was effectively simultaneous across the field of view. Note that the ∼3 ms time scale for thermal diffusion across the diameter of the laser spot is matched closely to the frame time, and thus, we expect the temperature rise to rapidly level off after the first few frames as continued laser-heating of the FOV is balanced by conductive heat loss to the environment. This consideration of thermal diffusion time scales accounts for the sharp temporal threshold (as rapid dewetting cannot occur until after melting), the spatial uniformity of the threshold, and the temporal uniformity of the motion after the threshold.

A great deal of information is present in the reconstructed video (Figs. 2 and S2 and Videos S2 and S3). The nanoparticle aggregates had already been sintered during previous similar exposures, such that the smallest ones had nearly reached their final nearly circular states, while the largest aggregates still had localized high-curvature regions that clearly evolved during the exposure. The general trend is the rapid motion of high-curvature regions in such a way as to reduce the curvature, and so the entire aggregate becomes more circular with time, as expected by a process driven by surface tension. The motion is decidedly liquidlike in visual appearance, with no coalescence of these already-sintered nanoparticles. This indicates that the process captured in this particular measurement truly is melting and dewetting rather than sintering.

The 100-frame 1.33-kHz CS reconstructed video of laser-driven sintering (Fig. 3) reveals a different physical process entirely. This time we consider a single isolated aggregate that had not been treated with previous laser pulses [Fig. 3(a)] and thus retained its initial nanometer-scale features including 33 20-nm-diameter irregular spheres and the gaps between them. Each of these gaps is a potential absorption hot spot, and we should expect the laser absorption per volume of such an aggregate to be considerably higher than that of a nearly fully dense single gold structure of comparable size^38^—as the nanoparticle aggregate has become clearly by roughly *t *=* *12 ms. Thus, in addition to the easily computed time scales in Table I (which plausibly account for the few-millisecond incubation time between the start of the laser pulses and the start of the sample motion), this process includes a new time scale emerging from the dynamics under study. Once the sintering has proceeded far enough to fill in the interstitial regions and eliminate most of the hot spots, the laser absorption should drop precipitously. Given the time scales in Table I and the expected higher absorption in the aggregate relative to the substrate, the temperature should rapidly drop as well as heat is lost to the cooler substrate, until the aggregate is too cold to support further sintering. The process is therefore inherently self-limiting under the tested conditions. The laser intensity in this experiment was sufficient to initiate sintering in the nanoparticle aggregate but insufficient to melt the sintered cluster.

The reconstructed video is easy to understand with this interpretation in mind. Note the distinct contrast between and within nanoparticles in the bright-field TEM image. This is in part caused by diffraction contrast, indicative of variations of crystal orientation, local thickness, and strain. This contrast is distinct from mass-thickness contrast responsible for a lightening of the edge of each nanoparticle relative to its interior and is specifically indicative of crystalline solid material as opposed to liquid. After a short ∼2 ms incubation time during which the aggregate is gradually heating, the temperature of the aggregate apparently reaches a threshold in frames 10–11, with a very rapid, single motion of several nanometers occurring simultaneously about most of the perimeter of the aggregate, combined with the simultaneous densification and nearly complete infilling of the gaps that initially separated the nanoparticles. Yet, the distinct diffraction contrast between and within the individual nanoparticles remains throughout the video, indicating that most (perhaps all) of the original nanoparticles never fully melted. Thus, the process is truly laser-driven sintering as opposed to melting, possibly enabled by enhanced surface mobility at temperatures that are elevated but still below the melting point. While we have no way of observing whether the fast-moving gold surface regions are liquid or solid, we can still affirm that most of the volume of most of the nanoparticles remained solid throughout the process, due to the persistence of the diffraction contrast in each grain. The evolution was essentially complete within 10 ms after the laser began firing, even though the laser continued to strike the sample through to the end of the 75 ms span. In fact, there is almost no sample evolution in the video after roughly *t *=* *12 ms. As the infilling of potential hot spots is visibly nearly complete by roughly *t *=* *9 ms and the time scale for heat diffusion out of the laser spot is roughly 3 ms, this is entirely consistent with the self-limiting interpretation.

The coarsening experiment is different in that the sample is a contiguous gold film, and thus, the relevant diffusivity for all scales is that of gold. This means that the thermal equilibration time scale across the drive-laser spot diameter is comparable to the laser repetition period and roughly 3 times shorter than the 100 μs reconstruction frame time. Thus, despite the 10 kHz frame rate, this experiment is still in the regime in which the temperature in the field of view should reach nearly the steady state (with the incoming heat balanced by heat thermally diffusing away from the laser spot) in just a few frames of video. Note that the laser takes roughly 5 frames to ramp up to its steady-state power as well. Thus, we expect a rapid temperature transient roughly from *t *=* *600 μs (frame 7, the onset of the laser drive) to *t *=* *1200 μs (frame 13, allowing for the 500 μs ramp-up and roughly 5 times the time scale for thermal diffusion across the laser spot), after which the temperature should level off and be roughly constant as a function of both time and space.

Despite the low contrast and signal-to-noise ratio in the 10 kHz reconstructed video, the results still tell a clear story that can be interpreted in terms of the classical theory of normal grain growth in thin films (as reviewed, for example, by Thompson^40^). The as-deposited structure [initial frames in Fig. 4(c)] is dominated by small (∼20–30 nm diameter) features with very weak image contrast. The coarsened structure [Fig. 4(d) and final frames in Fig. 4(c)] produce much stronger contrast under the same imaging conditions, with most grains having clearly defined sharp edges. Knowing the film to be 50 nm thick and knowing that these are bright-field diffraction TEM images that can be interpreted as projections through the 50 nm thickness, the difference has an obvious interpretation: the initial structure likely has a great many nearly equiaxed grains that overlap in the through-thickness projection, while the final structure is much more columnar, with most grains spanning the entire thickness of the film and most grain boundaries oriented nearly perpendicular to the film surfaces. The classical theory then predicts exactly what we observe: an initial burst of rapid grain growth followed by stagnation as the typical grain sizes reach roughly 100–150 nm (i.e., 2–3 times the film thickness) and the grain boundary network transitions from three-dimensional to two-dimensional.^40,41^ As was long ago noted,^42^ this dimensionality transition is insufficient to explain the suddenness of the onset of grain growth stagnation, and additional mechanisms must be involved such as the formation of stable grooves where columnar grain boundaries meet the thin film surface. The onset of stagnation in terms of the sizes of visible grains is remarkably quick and global across the field of view. The set of grains visible in frame 16 bears a clear resemblance to the set of grains in the final structure. Yet, the sample continues to evolve, more subtly, throughout the 10 ms span of the reconstructed video. Most notably, the contrast continues to increase and the features become sharper. This change in contrast may be due to several factors such as reduced motion blur as the grain growth stagnates, reduced thermal diffuse scattering (which would imply an unexpected reduction in temperature despite the ongoing laser illumination), elimination of remaining defects and extremely small grains, solidification of transient thin liquid layers at grain boundaries, and/or a gradual alignment of the grain boundaries to be tangent to the electron beam as the material evolves toward a well-annealed columnar structure with mostly vertical boundaries. The shapes of the grains continue to evolve slightly in the last 8 ms of the laser-driven process. In any case, the results suggest that stagnation in terms of producing nearly the final grain size distribution and stagnation in terms of producing a stable, well-annealed, fully solidified columnar structure are two entirely different things taking place on two quite different time scales.

### Potential future developments of ES

In summary, we have shown how electrostatic subframing, in its various operating modes, can reveal the details of millisecond-scale processes at the core of nanoscale materials science. While conventional TEM studies can explore the initial and final states, the flexible multikilohertz capability enabled by ES allows the crucial in-between states to be captured, over a wide range of parameters that can be tailored to each experiment. Each of these three test cases—melting/dewetting, sintering, and grain coarsening—is meant as an example of what such a study could look like.

The ES technique is still very new, and a great many possibilities exist for its future development beyond these proof-of-principle results. The tests demonstrated so far focus on *in situ* experimentation, but this is only one of many potential application areas. 4D-STEM is a compelling application, given the need for very fast frame rates, low single-frame pixel counts, and very high timing precision.

We have provided a proof-of-principle example of distortion correction, superposition, and optimal denoising of dose-fractionated images [Fig. 4(d)] of nominally static materials. Such images can be corrected for rigid full-field drift with existing reconstruction algorithms, and relatively modest development should allow generalization to include nonrigid dewarping of each individual subframe into a common reference frame. ES allows precise and flexible dose fractionation down to the submicrosecond regime.

Of course, compressive sensing can only use the information available to it, and it can actually perform quite poor if high compression ratios are attempted on measurements with a great deal of Poisson noise.^29,43^ Ultimately, beam current, beam brightness, and intrinsic sample contrast limit the spatiotemporal resolution in any time-resolved TEM investigation,^44^ and there comes a point where an experiment requires a much brighter source than is available. But the system we have described, with its wide range of operating modes and parameters (1-to-1 mode with and without the overlap resolution, full CS mode at varying time slice counts, aligning and denoising dose-fractionated images, and subframe modes from 2 × 2 to 16 × 16), provides new ways to make better use of all available information, tailored to the needs and challenges of each experiment.

## MATERIALS AND METHODS

### Hardware

To implement the temporal measurement matrix *M* in hardware, we require the deflection system to be fast, precise, highly programmable, and free of blur, hysteresis, and overshoot. The deflector needs to be able to change states multiple times per time slice to distribute the time among the subframes as dictated by *M*.

Our solution is a two-dimensional electrostatic deflector derived from the design for the movie mode dynamic transmission electron microscope (MM-DTEM).^4^ Here, we describe the implementation on the SNL I^3^TEM, a heavily modified JEOL 2100.^31^ Four deflector plates are placed below the projector lens, in two pairs acting as parallel-plate deflectors in the X and Y directions. Each plate is individually controlled with its own driving circuit. Each of the four circuits includes its own four programmable DC voltage supplies with variable output ranges spanning −420 V to +420 V. Each output stage allows any one of its four DC supplies to be connected to its output at any time. The switching times among the four voltage-on states are dominated by the RLC time constants of the output stage and the load, and the circuit is tuned to essentially eliminate overshoot. Based on oscilloscope measurements, we estimate the transition to be essentially complete within ∼20 ns. Thus, for millisecond-scale experiments, we can approximate the deflector as switching states instantaneously; the “blur” electrons are so few that we may take them to be part of the background noise. In practice, we observe no evidence of drift, blur, overshoot, or hysteresis on the TEM camera, even when switching at an average rate of ∼100 kHz.

Because each of the four deflector plates can be switched among its own set of four voltages, the system can produce arbitrary sequences selected from 4^4^ = 256 distinct deflector-on states, in addition to the deflector-off standby state. We use a subset of these states for a given experimental configuration. Each of the 16 voltages (4 DC supplies on each of the 4 channels) can be individually programmed, allowing the deflector driver circuit to be configured to subdivide the camera into subframe arrays ranging from 2 × 2 to 16 × 16. We performed most of the tests in 4 × 4 mode, which allowed the subframes to cover nearly all of the camera with a relatively little overlap [Fig. 1(d)].

The deflector driver is controlled by a 16-bit 125 MHz digital pattern generator (Spectrum M2i-7010-exp) operated using custom control software written in the commercial software MATLAB. The software allows the user to select the deflector mode (e.g., 4 × 4-subframe mode), the measurement matrix generation algorithm and parameters (e.g., 1-to-1 mode or Bernoulli random for CS mode), and timing parameters such as exposure times, trigger delays, and sacrificial-frame settings. The pattern generator is triggered by the camera controller, thus synchronizing the deflection to the exposure. 8 of the pattern generator channels are dedicated to selecting among the 256 deflector states, while the remaining 8 can be used for synchronizing external equipment, such as a pulsed laser directed at the sample or a triggerable *in situ* sample holder system. The external triggers may be programmed arbitrarily on the 8 ns sample clock, thus providing precise and detailed synchronization of the TEM data acquisition with external hardware.

Because the TEM's built-in beam blanker can take ∼5–15 ms to restore the full beam current and because the electron beam is significantly aberrated during the several milliseconds that the beam blanker is partially on, the software provides an option to use the electrostatic deflector as an auxiliary, high-precision, postsample beam blanker. One of the 256 deflector states is designated as the beam blanker state. Ideally, the beam blanker state deflects the beam entirely off the camera. However, for large cameras, the maximum deflector driving voltages may not suffice, and we choose one of the subframes (usually the upper-left) to be a sacrificial beam-blanker subframe [Fig. 1(d)]. The deflector is set to this subframe position and held there, while the TEM's built-in beam blanker is partially on. This is done at both the beginning and the end of the camera's exposure time, and the pixels covered by that subframe are automatically dropped from the analysis. When using the deflector as a beam blanker, the effective exposure time—the sum of the temporal measurement matrix elements—is therefore somewhat less than the exposure time specified in the camera control software. In tests on a static sample [Fig. 1(d)], we can see the sacrificial image and the 12-ms individual subframes simultaneously, and it is clear that aberrations from the TEM's built-in beam blanker are major limitations for conventional millisecond-scale exposures. The sacrificial image suffers from anisotropic blurring, while the other subframes are sharp, high-contrast, and feature-rich. These 15 subframes indicate what 12-ms exposures of nanocrystalline material should look like in a standard thermionic TEM, if the limitations of the beam blanker can be bypassed.

The deflector distorts the image somewhat, as close inspection of Fig. 1(d) will reveal. Each subframe has slightly different anisotropic magnifications, varying by typically a few percent and varying slightly with the position within a subframe. This effect is a distortion, not a blur, and thus, it can be calibrated and corrected. We calibrate the distortion by performing what we call an “alignment series,” which consists of a series of images with only one subframe in each image, including a final image with the deflector turned off. Using standard image segmentation methods (threshold, choose the largest region, fill holes, and optionally dilate and/or erode), we determine which pixels are exposed in each subframe while rejecting noise. We perform feature matching to align unique features in the deflected and undeflected images.

Specifically, we use Akaze^45^ to detect and describe local features. We then follow standard computer vision techniques including estimating candidate matches among deflected and undeflected images, model fitting for a random subset of matches, and use of RANSAC to identify and discard outliers among detected matches^46^ to find a final model describing a smooth quadratic warping function between the deflected and undeflected images. This requires an appropriate choice of sample region, with multiple unique high-contrast features across the field of view, but in practice, we have found this easy to achieve with a variety of samples. The typical RMS fit residual is less than 1 pixel. This is repeated for each subframe.

The results from segmentation and alignment are stored in what we term an “alignment solution,” which is used in the reconstruction analysis as described below. The alignment solution specifies, for each subframe, (1) which camera pixels are included in the subframe, (2) which pixel each camera pixel corresponds to in a single common undistorted reference frame, and (3) the Jacobian determinant at each pixel, needed for enforcement of local intensity conservation.

### Data analysis: General

CS reconstruction algorithms require us to process our measurements and experimental parameters into particular mathematical forms. In our case, we will be solving the following equations:

$$\begin{array}{c} \hat{x}=\underset{x\geq 0}{\text{argmin}}{\left|\mathrm{Wx}\right|}_{1,}\\ s.t.\;{\left|\mathrm{Ax}-b\right|}_{2}^{}\leq \epsilon . \end{array}$$ (1)

In these equations, *x* is a candidate reconstruction (a vector collapsed from a 3D hypermatrix with spatial indices *i* and *j* and time slice *t*), $\hat{x}$ is the final reconstruction, *W* is the matrix representation of the sparsifying transform, *A* is a scaled measurement matrix to be described below, *b* is a vector related to the measured intensity *y* at each pixel on the camera, *ϵ* is a specified positive constant, |·|_1_ represents the *l*_1_ norm (the sum of the absolute values of the vector elements), and |·|_2_ represents the *l_2_* norm (the usual Pythagorean length of a vector). This formalism, including the optional non-negativity constraint on each element of *x*, is directly implemented in the freely available software package TFOCS,^47^ and this is exactly what we used for most of the results in this article. In short, the optimization problem is looking for the reconstruction that is (1) non-negative everywhere, (2) consistent (within calibrated error) with the scaled measurement *b* and our understanding of the measurement process encoded in *A*, and (3) maximally sparse in the representation specified by the *W* matrix.

It takes significant decision-making, preprocessing, and calibration to get our real-world data in the form of Eq. (1). We will start with the sparsifying transformation *W*. The literature reveals that, for a great deal of image and video data, surprisingly good denoising and CS reconstruction performance is achieved using some version of a “total variation” (TV) penalty.^33,34^ In the present work, which is concerned with reconstruction of video data, we will use an anisotropic TV penalty defined as

$$\mathrm{TV}\left(x\right)={\left|\lambda_{s}\nabla_{i}x\right|}_{1}+{\left|\lambda_{s}\nabla_{j}x\right|}_{1}+{\left|\lambda_{t}\nabla_{t}x\right|}_{1},$$ (2)

where ∇_(__*i,j,t*__)_ is the discrete gradient in the specified direction and the positive scalar coefficients *λ_s_* and *λ_t_* govern the strength of the penalty in the space and time directions, respectively. This is implemented by defining the W matrix as a concatenation of the discrete gradient operators,

$$W=\left[\begin{array}{ccc} \lambda_{s}\nabla_{i} & \lambda_{s}\nabla_{j} & \lambda_{t}\nabla_{t} \end{array}\right].$$ (3)

The question remains of how to assign *λ_s_* and *λ_t_*. We can, without loss of generality, set *λ_s_* = 1 since in the original equation (1), a rescaling of *W* has no effect on the problem definition (it also has no effect on the numerics since TFOCS includes automatic preprocessing that compensates for the norm of *W*^47^). Thus, we set *λ_s_* = 1 and we vary *λ_t_*. We have not yet found a robust, objective way to set *λ_t_* (though theoretical work suggests some possible approaches^48^), and we adjust it freely so as to optimize the subjective appearance of the result. However, we have found that for a given reconstruction, there is typically a broad range of *λ_t_* values that produce substantially indistinguishable results. Intuitively, this makes sense: the purpose of minimizing the *l*_1_ norm is primarily to induce sparsity in *Wx* and not to penalize the magnitudes of its nonzero elements, and much of the theory of CS is based on the insight that the *l_1_* norm generally fills this purpose quite well (with some interesting exceptions^48^). We also note that the optimal *λ_t_* may depend on the purpose of the experiment. This parameter governs a contribution to denoising that uses the immediately preceding and following frames to determine whether a feature in a single frame is likely to be real. This denoising effect substantially reduces the apparent noise level but can come at the cost of reduced time resolution, as information can thereby bleed from one temporal frame into its neighbors. For applications demanding the absolute highest time resolution regardless of the signal-to-noise ratio cost, one should set *λ_t_* to zero. For the results presented herein, we did not do this; we adjusted *λ_t_* to achieve a subjective compromise of denoising and unambiguous reconstruction while ensuring that we saw no negative-time artifacts (i.e., apparent sample motion before the sample drive laser started hitting the sample), accepting that this may imply a loss of some very fast-changing details.

### Statistical analysis

Next, we need to interpret the |*Ax*-*b*|_2_ ≤ ϵ constraint in a way that makes sense given how the measurement works. Naively, we would set *b* equal to the image *y* on the camera and construct the linear operator *A* that models the camera image, given a reconstruction estimate. We can do this given our temporal measurement matrix *M* (showing how the set of time slices, i.e., reconstructed frames, gets superposed into the set of measured frames) and the alignment solution (showing how the set of measured frames gets mapped to the camera, including overlaps). Taking the appropriate tensor inner product of these two entities produces a matrix *A*^0^, such that *A*^0^*x* is an estimate of what would be measured on the camera and which needs to be compared with the actual measurement *y*.

However, this ignores the noise characteristics of the camera, such that the error in the signal is strongly correlated with its magnitude. This needs to be calibrated, yielding an estimated noise vector *σ*, the same size as *y*. This allows us to construct a fully calibrated *χ*^2^ goodness-of-fit metric, following a standard error-scaling approach to allow least squares algorithms to properly account for different errors in each data point,

$$\chi^{2}=\sum_{i}{\left(\frac{\sum_{j}A_{\mathrm{ij}}^{0}x_{j}-y_{i}}{\sigma_{i}}\right)}^{2}.$$ (4)

We can then define *A* to be the result of dividing each row of *A*^0^ by the corresponding element of *σ* (i.e., $A_{\mathrm{ij}}=A_{\mathrm{ij}}^{0}/\sigma_{i}$, with no implied summation) and similarly *b_i_* = *y_i_*/*σ_i_*. With this ansatz, we immediately get *χ*^2^=|*Ax*-*b*|^2^ ≤ *ϵ*^2^.

Because we performed an absolute noise calibration, this gives us a way to objectively set the value of *ϵ*, at least to within a factor *κ* of order unity (introduced previously). Let *χ*^2^ = *ϵ*^2^ = *κN_valid_*, where *N_valid_* is the number of valid camera pixels (1.5 × 10^7^ for the tested 4096 × 4096 pixel camera with one sacrificial subframe out of 16). A “valid” pixel is one that is covered by at least one subframe but is not part of any sacrificial subframe. The error *σ* for invalid pixels is set to infinity so that they do not take part in the *χ*^2^ sum. Setting *κ* = 1 therefore specifies that we are looking for a solution where the rms error in the fit is exactly what you would expect from measurement noise (an approach closely related to the approach in the study by van den Broek *et al.*,^29^ though the formalism superficially looks entirely different). Indeed, in our reconstructions, the subjectively best-looking results were obtained near *κ* = 0.25–1, with the lower values tending to give better results at lower signal-to-noise ratios. Increasing κ increases the denoising, but at the cost of possible loss of real features. Generally, we found results with κ ∼ 2 or more to appear oversmoothed.

The camera noise calibration is done by collecting multiple images of uniform illumination in a range of known exposure values, a procedure very similar to the measurement procedure for the familiar dark/gain calibration used in most TEM cameras. For the SNL I^3^TEM data, we captured 72 repeats each at four different exposure times: 0, 50, 200, and 1000 ms. This allowed us to determine the means and standard deviations, across the ensembles of 72 images each, for the measured intensities at each pixel on the camera. An uncertainty weighted linear fit produced the familiar dark count and gain calibration images, allowing us to convert measured intensities into corrected intensities as is usually done for CCD TEM cameras. We then performed a weighted linear fit of the statistical variance $\sigma_{y}^{2}$ of intensity as a function of the corrected intensity *y*,

$$\sigma_{y}^{2}\left(y\right)=\alpha y+\beta .$$

In this equation, *β* is the variance of the dark noise, while *α* is a coefficient for a term that scales as Poisson noise. We found that the *α* term dominated except at exposure levels of one electron per pixel or less, and thus, a calibrated Poisson noise model is an excellent approximation throughout the useful exposure range. This is important, as it significantly affects the compression-distortion bounds and optimal measurement strategies for CS reconstructions.^29,30,43^ While our rescaling allows the reconstruction to be performed using a model that assumes additive Gaussian noise, the underlying data definitely do not follow such a noise model, and practical evaluations of the value of CS need to keep this in mind. σ in Eq. (4) is thus $\sigma_{y}(y)$, evaluated from the measured intensity *y* and not the modeled intensity *A*^0^*x*, which is the appropriate choice for minimizing bias in the implicit approximation to the Poisson likelihood function.^29^

In the original formulation [Eq. (1)], we have now accounted for *x*, *W*, *b*, *ϵ*, *σ*, the construction of the unscaled *A*^0^ measurement matrix, and the uncertainty-rescaling that transforms the matrix *A*^0^ into the *A* matrix used in the CS reconstruction. This fully defines the CS reconstruction problem. In practice, we find that we obtain the best performance when we first do a coarse reconstruction at a high binning level (combining 8 × 8 blocks of pixels, for example), thus allowing rapid determination of optimal algorithm parameters and permitting the algorithm to coarsely sort out which features and events belong to which frames. We can then software-unbin by a factor of 2 at a time and refine the solution.

The error estimate *σ* deriving purely from camera noise is insufficient at the edges of the subframes, largely because the edges of the subframes on the camera can only be calibrated to finite precision. Subtle changes in lens focus, alignment, and deflector positioning will all slightly shift the projector lens crossover relative to the deflector, and this can shift the subframe edges by several pixels on a large-pixel-count camera. To compensate for this, we do three things: (1) soften the subframe edges with an arctangent smoothing function derived from curve fits to the edges of the alignment-series subframe images, (2) determine a slight vector shift of each subframe on the camera using maximum cross correlation methods, and (3) increase the estimate of σ for all camera pixels within a specified distance of the edge of any subframe, thus expanding the definition of σ to include this source of systematic noise along with the random noise from the camera detection process. This helps to flatten the normalized-residual images and reduce the subframe-edge artifacts visible in the video reconstructions. We are endeavoring to further reduce this problem by (1) improving the mechanical stability and repeatability of the deflector positioning, (2) fine-adjusting the deflector geometry so that exactly the same sample field of view is visible in all subframes, and (3) simplifying and speeding up the alignment series measurement and analysis, thus making it painless to quickly perform an alignment series after any significant refocusing or realignment.

### Data analysis: Final details

There is one more correction to the *A* matrix that, while not strictly necessary, can improve the quality of the reconstruction, namely, drift correction. In many *in situ* experiments, the sample moves in the field of view during the process of interest. As the experiments often involve rapid localized heat, stress, or chemical reactions, this is nearly inevitable; it is part of the physics of the subject under study. The anisotropic TV penalty, as formulated [Eq. (2)], does not compensate for this; the temporal gradient ideally should be a local convective time derivative (i.e., comoving with the sample) rather than a partial derivative. A simple method to deal with this is to determine the global displacement as a function of time and compensate for it. This is easily accomplished in the construction of the *A*^0^ matrix from the measurement matrix and the alignment solution; each pixel in the undistorted source space simply needs to be shifted as a function of time during the construction. We can determine the drift by performing a rough reconstruction and finding the vector displacement vs time that keeps the features in the center of the field of view. We then adjust *A*^0^ and repeat the calculation.

This method results in a center-of-mass-stabilized video that helps to accentuate the actual dynamics of the material while suppressing the much less interesting global drift. Of course, this means that the edges of the visible field of view will shift throughout the reconstructed video, and this is apparent in the subframe-edge artifacts in our reconstructions. We elect to keep a larger-than-necessary output video size to ensure that we do not lose any real information. This means that some space-time voxels in the reconstruction are unconstrained by the measurement, and these can be removed at the end of the process as mentioned previously.

The reconstruction calculation itself uses the smoothed, weighted basis pursuit denoising algorithm implemented in TFOCS,^47^ with a non-negativity constraint, using continuation to accelerate convergence while minimizing the ultimate effect of the artificial smoothing function so that the algorithm ultimately converges on the solution of the original problem. As this is a standard use case for the well-documented open-source TFOCS software, we refer the reader interested in the numerical reconstruction algorithms to the documentation supplied with TFOCS. We first performed rough-draft reconstructions with spatial binning of 8 × 8-pixel blocks. This allowed us to quickly adjust the most important parameters (which turned out to be *λ_t_*, *κ*, the subframe-edge error-estimate enhancement parameters, the TFOCS artificial smoothing coefficient *μ*, and the estimated squared norm of the *W* matrix, which needed to be increased typically ∼10-fold from its actual value in order to keep the *ϵ* constraint satisfied) and see the effects on the reconstruction. Evaluation of the reconstruction performance is done by viewing diagnostic-tableau graphics such as in supplementary material Fig. 6, as well as viewing the reconstructed videos themselves.

## SUPPLEMENTARY MATERIAL

See the supplementary material for the explanation of the first proof of principle measurements at LLNL, all frames from all reconstructed videos including both directly measured and interpolated/extrapolated versions, photodiode measurements of the drive laser temporal envelope, and an example diagnostic image. AVI format videos of all reconstructions are also available.
